# Exploring beliefs around physical activity among older adults in rural Canada

**DOI:** 10.3402/qhw.v11.32914

**Published:** 2016-11-09

**Authors:** Laurie Schmidt, Gwen Rempel, Terra C. Murray, Tara-Leigh McHugh, Jeff K. Vallance

**Affiliations:** 1 Centre for Nursing and Health Studies, Athabasca University, Athabasca, AB, Canada; 2 Faculty of Physical Education and Recreation, University of Alberta, Edmonton, AB, Canada

**Keywords:** Rural, physical activity, older adults, health, socio-ecological model

## Abstract

**Objective:**

As physical activity can improve health and reduce the risk of chronic disease, it is important to understand the contributing factors to physical activity engagement among older adults, particularly those living in rural communities to assist in remaining active and healthy as long as possible. The purpose of this study was to gain a deeper understanding of the socio-ecological factors that influence or contribute to physical activity among rural-dwelling older adults in rural Saskatchewan, Canada.

**Methods:**

This qualitative description explored the perceptions of physical activity among older adults living in two rural communities in the Canadian province of Saskatchewan. Semi-structured interviews were conducted with 10 adults aged 69–94. Using content analysis techniques, transcribed interview data were coded and categorized.

**Results:**

Participants identified socio-ecological elements facilitating physical activity such as improved health, independence, and mobility as well as social cohesion and having opportunities for physical activity. The most common perceived environmental barrier to engaging in physical activity was the fear of falling, particularly on the ice during the winter months. Participants also cited adverse weather conditions, aging (e.g., arthritis), and family members (e.g., encouraged to “take it easy”) as barriers to physical activity.

**Conclusion:**

Hearing directly from older adults who reside in rural Saskatchewan was determined to have the potential to improve awareness of physical activity in rural communities to support the implementation of programs and practices that will facilitate active lifestyles for older adults.

Not only is Canada’s population increasing, it is also aging. Canadians aged 65 years and older are the fastest growing age group. By 2036, the number of older adults age will increase to between 9 and 10 million, which is more than double the older adult population measured in 2009 (Statistics Canada, 2012). By 2021, the number of older adults is expected to surpass the number of children aged 14 and under for the first time in Canadian history (Statistics Canada, 2012). In 2009, 89% of older adults had at least one chronic condition (Public Health Agency of Canada, 2010).

Physical activity is an effective intervention to enhance the overall health, quality of life, reduce the risk of falls, and reduce risks associated with chronic conditions among older adults (Health Canada, 2011). In addition, being physically active also improves physical and mental functioning, social well-being, and overall physical and physiological health. Particularly among older adults, physical activity improves and maintains strength, flexibility, balance, and coordination as well as reduces risk of falls. Older adults are the least physically active age demographic (Colley et al., 2011). Objectively assessed estimates of physical activity suggest that Canadian older adults (aged 60–79) accumulate only 17 min of moderate-to-vigorous physical activity (MVPA) per day (Colley et al., 2011). Only 13% were accumulating at least 150 min per week of MVPA (as per public health guidelines). Little research has been conducted on physical activity among rural-dwelling older adults in Canada, particularly in the Prairie Provinces.

In Canada, 23% of older adults reside in rural communities and for some rural areas this number is increasing (Hamilton, 2008). Even though there are benefits to living in rural communities (e.g., having a greater sense of community and volunteerism) (Graham & Connelly, 2013), older adults living in rural communities often report poorer health outcomes such as increased respiratory disease, injuries, and suicides and the impact of chronic disease is greater, compared with those living in urban centers (Canadian Institute for Health Information, 2006). Older adults living in rural communities also report limited income, education, and higher incidence of unhealthy behaviors such as smoking and obesity (Canadian Institute for Health information, 2006). Given these multiple factors, it is important to consider the interaction between personal, social, and environmental elements that influence health, particularly among older adults in rural communities where health-promoting behaviors such as physical activity remain low. To our knowledge, only one study has examined physical activity among older adults in the Canadian maritime rural context (Fogo Island, NLD) (Witcher, Holt, Spence, & Cousins, 2007). Witcher and colleagues found that the context in which participants grew up affected their physical activity participation. People who had a physically demanding career as a young adult had little time or interest to engage in leisure-time physical activity particularly into older adulthood. They also reported that those who were active in older adulthood conducted what they perceived as meaningful physical activities such as berry picking, wood gathering, knitting, and sewing.

In 2007, the province of Saskatchewan had the highest number of older adults (≥65 years of age) in Canada which has since reduced to just above the national average. However, Saskatchewan is one of the last provinces in Canada that is yet to implement a strategy on healthy aging, and little research has been done on physical activity among older adults in rural communities, particularly using a socio-ecological framework. For this study, we used qualitative description to explore the perceptions of physical activity among older adults in rural communities in Saskatchewan. Specifically, the purpose of this study was to gain a deeper understanding of the socio-ecological factors that influence or contribute to physical activity among rural-dwelling older adults in Saskatchewan.

## Methods

Qualitative studies aim to gather the perspective of individuals and to experience meaning, situations, and actions based on their perceptions (Hallberg, 2013). For the purpose of this study, the qualitative research method of qualitative description (Sandelowski, 2000, 2010) was chosen as it enabled the researcher to identify and understand the interaction of influences on physical activity among older adults in rural Saskatchewan. Qualitative description was used to gain a deeper understanding of the perceptions of physical activity among older adults in rural Saskatchewan. Qualitative description is useful when little prior knowledge exists in a particular area (Ceria-Ulep, Serafica, & Tse, 2011). This approach generates findings that can be used in creating or revising interventions, conducting pilot interventions or developing programs (Sullivan-Bolyai, Bova, & Harper, 2005). Qualitative description produces a straightforward, in-depth description of participants’ experiences in words as similar as possible to what the participants say (Sandelowski, 2000). The research question is usually a simple question that is used to gain insight into a participant’s experience within a specific topic area (Magilvy & Thomas, 2009). Similar to other research methods, the research question in qualitative description studies guides the entire study (2009). Theory within this approach often appears in the background knowledge of the researcher through experience, knowledge gained in the topic area, and through reviewing the works of others in the field (Sandelowski, 2010). Qualitative description ensures that information represents the perspective of the participant, not the researcher, which is valuable in gaining an understanding and hearing the voice of those affected (Sandelowski, 2000). This study was approved by the Athabasca University Research Ethics Board (#1324-24).

### Communities and participants

Qualitative studies demand data from relatively small and strategically selected study groups (Hallberg, 2013). Aligning with recent research on rural and remote communities in Saskatchewan, the definition of rural for this study was based on Statistics Canada’s definition where rural and remote areas consisted of a population of less than 10,000 people and a population density of up to 400 people per square kilometer (Saskatchewan Health Research Foundation, 2007). Recruitment of participants focused in two rural communities. One community was located in the Southeast corner of Saskatchewan and was designated a town in 1904 and had a current population of 1285 people (Statistics Canada, 2012). Due to the established recreation opportunities and contact people available, this community was the primary town from which the participants were recruited. A neighboring community smaller in population (342 inhabitants) but similar in services was also used for recruitment.

To gain in-depth information from participants, purposive sampling (Glesne, 2011) was used to recruit 10 older adults in two rural communities in Saskatchewan. According to Creswell (2013), there are three considerations in choosing the purposive sampling approach that include who to select as participants, what type of strategy will be used, and how many participants will be selected. Specific sample sizes have not been identified for qualitative description studies (Sandelowski, 2000) and, as such, participants were recruited until the data reached a level of saturation (i.e., no new information was observed). Recruitment was facilitated by health care and recreation staff with extensive knowledge of the community. Inclusion criteria consisted of adults aged 65 and older, English-speaking residents of the community, and either physically active or inactive individuals. Potential participants were contacted by telephone by the researcher and the study explained. The researcher confirmed participant eligibility. Participants gave informed consent to participate and were given a $20 gift card to a local retailer to compensate for their time.

### Data generation

Data were generated between March and April 2015 from 10 participants by way of individual semi-structured interviews (by telephone and in person) lasting approximately 30 min in duration. Engaging in individual interviews, participants described their experience with little interference by the researcher in guiding or leading the discussion. A semi-structured interview guide was used to maintain focus on the research question (e.g., what are some benefits you get from being active?; where in the community are you physically active?; what stands in the way of you being more active?). Open-ended questions invited the participant to describe their experience with little interference by the researcher in guiding or leading the discussion, and semi-structured interviews allow for open-ended conversations while remaining focused on the research question (Sandelowski, 2000).

Observational/contextual data were generated and recorded through a search and review of the town website, and publically accessible town council minutes that recorded infrastructure projects, resident concerns, and planning for town services and events. Such data allowed the researcher to become familiar with the services and opportunities that existed in the communities (Neergaard, Olesen, Andersen, & Sondergaard, 2009). These data were mentally recalled during interviews by the researcher to assist in bringing to life the setting and description put forth by participants. These data also assisted in developing a broad understanding of the participant’s experience within their community and assisted in providing a reference point when participant’s described their perceptions. As a means of gathering contextual data and ensuring rigor in the study, both descriptive and reflexive notes were kept within a research journal. These journal notes were reviewed during the data analysis to maintain awareness of potential interference and served as a means of rigor in the study by identifying researcher bias, influences, and contextual information (Creswell, 2013). Descriptive notes recorded observation data.

Reflexive notes were made during and after each interview in a journal documenting initial thoughts, impressions, and questions that came to mind while engaged with the participant. Also noted were certain data that touched on the researcher’s own personal experience with older adults. These reflexive journal notes were reviewed during the data analysis to maintain awareness of potential interference and served as a means of rigor in the study by identifying researcher bias, influences, and contextual information (Creswell, 2013). For example, when a participant was describing physical activities that she was still involved in, a reflexive note was made that the researcher mentally compared the strenuous activity and age of the participant to a family member similar in age and activity to the researcher.

### Data analysis

Content analysis was used as outlined by Elo and Kyngäs (2008). Content analysis is an approach of data analysis that is often used when a clear description is preferred by the researcher over an interpretation of the data (Vaismoradi, Turunen, & Bondas, 2013). There are three key stages to qualitative content analysis that include preparation, organization, and reporting of data (Elo & Kyngäs, 2008). Interviews were audio-recorded, transcribed verbatim, and analysed for common categories using manual techniques in addition to the software NVivo10 (QSR International, Burlington, MA) to help sort and store the data in a retrievable and searchable format. Transcripts were reviewed, immersing the researcher in the data. Notes were made in the margins with each read including general impressions of main concepts for each participant. Emerging code categories were documented on a separate sheet, color coded, and defined according to participant’s own descriptions. Once defined, the process of categorizing began. Once all codes were reviewed in reference to the original data, categories were formed. The researcher repeatedly referred to the descriptive and reflexive notes when making decisions to group codes. In addition to manual analysis, interviews and code categories were uploaded to NVivo10 that assisted in sorting and referencing data generated in the study.

## Results

### Participants

Data were provided by 10 older adults that comprised 9 females and 1 male ranging in age from 69 to 94 years of age (*M*=78.8; *SD*=9.3). Of the 10 participants, 6 were widows and 4 were married. Nine participants lived independently and in their own home with one living in a supportive care home. All participants were retired from their occupations; however, most continued to volunteer their services in the community. To protect the anonymity of study participants, details about individual participants have not been associated with the data they provided in the writing of the results. A pseudonym has been assigned to each participant and all participants are referred to as females to protect the one male in the study. Pseudonyms were chosen from listings of top names for the years the participants were born.

### Maintaining health

A key facilitator of physical activity that emerged among participants was to maintain health. Participants noted both physical and mental health benefits as a result of being physically active and cited physical activity as contributing to living independently in their own homes for as long as possible. Helen (age 90) summarized the view of the study participants about the benefits of physical activity: “I feel good. It limbers up the old joints. You sleep better and then you’re hungry.” Study participants described their desire to maintain physical mobility as a motivator for staying active. Physical activity helped participants keep supple enough to be able to do the things they wanted to. The necessity for regular activity was emphasized by many of the participants and according to Betty (age 85) “I miss it if I am not able to, don’t go (to exercise class) for a few days. I really go stiff.”

### Social hour

In addition to maintaining physical and mental health, participants placed value on social interactions with others in their community, including their friendships, being known in the community, their volunteer work, and their family. These all served as important facilitators to participating in both intentional physical activity as well as daily activities. Helen said, “I like to go (exercise) because I am alone and it’s good to go and it’s a social hour.” Shirley (aged 77) said “I like the opportunity you have to be busy and then to have people you know and to, you know, be recognized on the street and, that’s the part I like about it I guess.”

### Volunteer opportunities

The participants in this study recognized the importance of volunteering in the community and spoke about their role and responsibility to help out at a number of organizations including the church, the care homes, and within the town itself. Volunteering provided a reason to get out of their homes and be active in their community. Shirley noted that “Naturally the older people are considered an important part of the community” however as stated by Dorothy (aged 94) “You have to be dedicated. You have to. You have to work.” More than half of participants articulated the importance and their gratitude for having their family live close by. Many noted that family was a key facilitator in their current physical activity level. Judith (age 86) stated that it was nice to have her grandchildren stop in after school. She said “It was nice to have them around. They did a bunch of chores for me.”

### Accessible and safe

Social facilitators were closely related to environmental influences where the rural setting contributed to community accessibility and safety. Ruth (aged 76) stated “Everybody is behind you; everybody encourages you to do the best you can. We live in a wonderful place and I think we are lucky to be [here].” Participants noted that there were numerous opportunities to be active within their community. For Judith, the services she needed were within walking distance in her rural community and she could easily get to and from on her bicycle. “No sense starting the car or walking when I can get there faster on the bike.” For Betty (aged 85), walking to conduct errands was a common daily activity as she lived close to facilities such as the post office and senior’s center. She stated, “I live close to downtown and I walk where I can.”

Questions presented to participants were also intended to explore the socio-ecological barriers of their engagement in physical activity and included personal, social, and environmental elements.

### Aging

Many participants spoke about physical limitations as their bodies continued to age and noted the effect on their ability to be physically active. “My arthritis determines how much I can actually do because when I am in pain I stop doing whatever it is. I won’t push it” (Dorothy). Patricia (aged 69) spoke poignantly about some of the difficult aspects of aging, “I’ve learned it’s all part of what happens; you have to come to understand that, oh my goodness, I’m not going to be able to do the things I used to do.” A fine balance existed between remaining as engaged as possible with life, realizing limitations, and readjusting and compensating as Joan conveyed “I find it takes me longer to do things. I have a bit of arthritis and I just try to ignore it and pretend it’s not there and I think that the stretching exercises helps.” As Betty succinctly stated, “We have this saying, you do what you can.”

### Concerned family

Socially, participants conveyed that often their close family members impeded their physical activity participation by placing limitations on their opportunities for independence. For others, having family members living elsewhere also presented a barrier as they had less reason to engage in community activities and functions when their family was not involved. Carol (aged 69) explained that “I haven’t been to the rink in a long time because when your kids are gone you just don’t do that sort of thing anymore.” Even though in some cases, having family close by facilitated physical activity by encouraging older adults to get out and get active, for participants like Judith, having family closely involved also led participants to describe restrictions and cautions suggested by their family members. “I have a son that lives down here not too far away which is of benefit for me but he won’t let me drive to the city anymore but I guess maybe that’s okay” (Judith). She went on to explain that she was once very involved in numerous volunteer roles in the community that she had now given up: “My kids keep telling me, you’re not 59 anymore.” Likewise, Dorothy shared a similar position regarding her family, “My sons are naturally concerned. They are overly concerned about my health.”

### Fear of falling

The most commonly perceived environmental barrier to engaging in physical activity was the fear of falling, particularly on the ice during the winter months. Judith spoke of a friend who had fallen on ice and broken a hip and admitted, “I’m oversensitive about falling because, you know.” As a result, Judith now questioned her continued involvement in other activities such as winter curling. Joan (aged 73) revealed, “When it’s icy you really don’t want to fall” which kept her from being physically active. Dorothy admitted “I won’t even go out if it’s icy unless I have to.” The cold winters also brought awareness of a lack of opportunities for older adults to be safe and active during the winter months. It was during the winter seasons that these older adults perceived a lack of opportunity to safely engage in physical activity. Joan stated, “Right now we can’t walk, it’s too icy and mucky and slushy.” Many participants stated that an indoor walking facility would entice them to continue to walk though out the winter months. “There is a gym downtown but I find it very expensive and I have to find time to go there. On a limited income you have to figure out whether you want to spend your money there or somewhere else” and “It would be nice to have a bigger place … that’s not so expensive to walk in. Definitely (cost) is an issue” (Joan). Similarly, Barbara (aged 69) explained, “I would like to see a room or a building or whatever would allow indoor walking in the winter months … because our streets were atrocious and I have already fallen and broken bones.”

## Discussion

The purpose of this study was to explore the perceptions of physical activity among older adults in rural Saskatchewan in terms of personal, social, and environmental elements using qualitative description. Older adults living in rural Saskatchewan identified socio-ecological elements facilitating physical activity such as improved health, independence, and mobility as well as social cohesion and having opportunities for physical activity. Older adults from this rural region citied adverse weather conditions (e.g., fear of falling), aging (e.g., arthritis), and family members (e.g., encouraged to “take it easy”) as barriers to physical activity. The data generated from the study provide an overview of facilitators and barriers of physical activity that many older adults in rural Saskatchewan communities may experience. Both facilitators and barriers were reported across the socio-ecological sectors including personal, social, and environmental influences.

Participants also indicated that social elements facilitated their physical activity behavior. Having connections with friends and the community through volunteer opportunities, socializing as part of organized programs and activities, in addition to close family connections all contributed to physical activity engagement among study participants. Family was an important motivator for many participants who indicated that their family encouraged them to get out and be involved in their children or grandchildren’s activities. Often, this gave participants a reason to be sociable and interact with their family and community. The finding that social elements facilitated physical activity is consistent with previously published research. Research findings from 40 rural-dwelling older adults by Bacsu et al. (2014) found many older adults valued their volunteer opportunities within the church and the community for the social interaction they received. Further, Hand, Law, Hanna, Elliott, and McColl (2012) systematically reviewed 689 articles regarding neighborhood influence on physical activity participation among older adults with chronic health conditions. Of these, 15 studies indicated that older adults living close to friends, family, and social networks had higher incidence of physical activity engagement supporting the facilitating social element of physical activity. More recently, in a qualitative study by Kim, Yamada, Heo, and Han (2014), older Korean adults (involved in sports clubs) found social support was a key theme that emerged in the data. Together, these studies reinforce that older adults perceive socialization among family, friends, and their community as an essential factor in physical activity participation.

It is important to recognize the value placed on social cohesion among rural-dwelling older adults to support and reinforce their continued activity. Living in a safe and supportive environment facilitated physical activity among older adults in this study. Participants noted that they received a lot of community support, which they indicated was very important to them. Living in a community that they perceived as safe was also valued. Living in a community with safe roads, clear sidewalks, good lighting, and numerous facilities and services also encouraged participants to be active. Previously published evidence supports the facilitating elements of safety and support. Chrisman, Nothwehr, Yang, and Oleson (2015) conducted three focus groups among 19 rural residents and concluded that safe communities, low crime rate, as well as low traffic rates, influenced physical activity. Chrisman et al. also reported that older adults living in a perceived safe neighborhood were twice as likely to be physically active. Overall, it is evident that the physical and built environments contain important facilitating elements of physical activity engagement among older adults. Knowing the benefits of various elements in the community is useful in future and existing town planning and design, making note of modifiable factors such as keeping roads and walkways clear to increase safety and prevent unnecessary falls.

Participants discussed aspects of their environment that inhibited their activity such as adverse weather conditions that produced ice, slush, or strong winds making physical activity a less-desirable option. Overwhelmingly (9 out of 10), older adults in this study indicated a fear of falling, particularly when conditions are less favorable. The fear of falling related to inclement weather is abundant in the literature, particularly among the older adult population. Throughout the literature, the experience of falls and falling is intricately linked to activity. For example, in one recent qualitative study of older adults, Clancy, Balteskard, Perander, and Mahler (2015) concluded that in the experience of falls and falling, “participants’ stories reveals life courage and endurance and is more about getting up, staying up, and moving on than falling down.” (p. 9). Several studies have reported that older adults perceived adverse weather including ice, cold, snow as well as extreme heat as barriers to participating in physical activity (Chrisman et al., 2015; Marquez et al., 2014; Witcher et al., 2007). Also related to weather in rural communities, Chrisman et al. (2015) found that loose gravel and muddy roads inhibited physical activity among adults in the rural Midwest USA. This supports the data generated in this study about the cautious approach to being active in rural Saskatchewan when the road conditions were perceived as unstable. As weather was a main barrier for most participants in the study, it is important for communities to search for means to accommodate older adults during inclement weather, particularly throughout the winter months by creating or adapting appropriate and publicly accessible facilities and programs without cost where older adults will feel confident to attend and participate.

In addition to weather, the process of aging itself was a perceived barrier to physical activity for many older adults in this study. Many reported arthritis and other joint pain as limiting their ability to do the things they wanted to do. However, some were able to identify ways that they compromised for their situation. Jancey, Clarke, Howat, Maycock, and Lee (2009) reported that older adults reduced, and often stopped, physical activity when aches and pains were experienced. Many of Jancey’s study participants reported a loss of flexibility, balance, and lack of confidence in their abilities as barriers to activity. For some participants, when pain or discomfort limited their abilities, they accepted it as a part of aging, whereas others expressed their frustration over the situation. Gavarkovs, Burke, and Petrella (2015) reported that many rural-dwelling Canadian men indicated that pain or injury, in addition to being too tired, was a commonly mentioned barrier to physical activity engagement. Gaining a broader understanding of the personal health issues that many older adults face as they age is important when supporting activity and implementing preventive measures to reduce injury and illness.

Social barriers were apparent among some older adults in this study who spoke of family members’ perceptions of their ability to remain active, offering the suggestion that they slow down. Having family close by often encouraged older adults to get out; however, those with family that lived far away had little reason to continue accessing the local programs and services as they had no children or grandchildren involved to motivate or give them reason to partake in. Jancey et al. (2009) reported that some older adults experienced social stereotyping regarding age and ability. Some participants indicated that they were not socially supported to be physically active due to their age. Even though many older adults perceived family members as a supporting element in their activity, for some, family members also discouraged activity and independence by suggesting that restrictions be placed on specific activities as adults continue to age. Hearing directly from older adults who reside in rural Saskatchewan has the potential to improve awareness of physical activity in rural communities to support the implementation of programs and practices that will facilitate active lifestyles for older adults in the rural context.

There are several limitations and strengths of this study that deserve mention. Exploring the perspectives of participants among such a broad age range gave strength to the richness of the data generated; however, the sample size of 10 participants was relatively small and there was an uneven gender representation, so a rich description of older men’s perspectives was not generated. In addition, all participants were Caucasian and all perceived themselves to be physically active, leaving a gap in the data among the sedentary as well as the population of newcomers that resided in the communities. Although the participants who were already interested and motivated to participate in physical activity provided valuable insight, the perspective of the inactive segment remained unheard. The strengths of this study lie in the chosen methodology, the researcher’s own expertise, and the socio-ecological model on which the study was based on.

This qualitative description study of the perceptions of physical activity among older adults in rural Saskatchewan offers a greater understanding of physical activity among the older adult population within a rural context. Findings from this study help to gain insight into perceived facilitators and barriers in rural communities that older adults perceived as influencing their participation in physical activity. Identifying the personal, social, and environmental elements that facilitate and inhibit activity provides a more comprehensive understanding of older adult’s perceptions in rural Saskatchewan. Future programs and initiatives designed to increase physical activity participation among rural-dwelling older adults need to consider multilevel elements and their interactions to successfully promote and support physical activity.
